# *Salmonella enterica* serovar Typhimurium ST313 sublineage 2.2 has emerged in Malawi with a characteristic gene expression signature and a fitness advantage

**DOI:** 10.1093/femsml/uqae005

**Published:** 2024-03-28

**Authors:** Benjamin Kumwenda, Rocío Canals, Alexander V Predeus, Xiaojun Zhu, Carsten Kröger, Caisey Pulford, Nicolas Wenner, Lizeth Lacharme Lora, Yan Li, Siân V Owen, Dean Everett, Karsten Hokamp, Robert S Heyderman, Philip M Ashton, Melita A Gordon, Chisomo L Msefula, Jay C D Hinton

**Affiliations:** School of Life Sciences and Allied Health Professions, Kamuzu University of Health Sciences Blantyre, Blantyre, 265, Malawi; Institute of Infection, Veterinary & Ecological Sciences, University of Liverpool, Liverpool, L69 7ZB, United Kingdom; Malawi–Liverpool–Wellcome Programme, Blantyre, 3, Malawi; Institute of Infection, Veterinary & Ecological Sciences, University of Liverpool, Liverpool, L69 7ZB, United Kingdom; Institute of Infection, Veterinary & Ecological Sciences, University of Liverpool, Liverpool, L69 7ZB, United Kingdom; Institute of Infection, Veterinary & Ecological Sciences, University of Liverpool, Liverpool, L69 7ZB, United Kingdom; Institute of Infection, Veterinary & Ecological Sciences, University of Liverpool, Liverpool, L69 7ZB, United Kingdom; Department of Microbiology, Moyne Institute of Preventive Medicine, School of Genetics and Microbiology, Trinity College Dublin, Dublin, D02 PN40, Ireland; Institute of Infection, Veterinary & Ecological Sciences, University of Liverpool, Liverpool, L69 7ZB, United Kingdom; Institute of Infection, Veterinary & Ecological Sciences, University of Liverpool, Liverpool, L69 7ZB, United Kingdom; Institute of Infection, Veterinary & Ecological Sciences, University of Liverpool, Liverpool, L69 7ZB, United Kingdom; Institute of Infection, Veterinary & Ecological Sciences, University of Liverpool, Liverpool, L69 7ZB, United Kingdom; Institute of Infection, Veterinary & Ecological Sciences, University of Liverpool, Liverpool, L69 7ZB, United Kingdom; Department of Public Health and Epidemiology, College of Medicine and Health Sciences, Khalifa University, Abu Dhabi, P.O. Box 127788, United Arab Emirates; Smurfit Institute of Genetics, School of Genetics and Microbiology, Trinity College Dublin, Dublin, D02 PN40, Ireland; Malawi–Liverpool–Wellcome Programme, Blantyre, 3, Malawi; Research Department of Infection, Division of Infection & Immunity, University College London, London, WC1E 6BT, United Kingdom; Malawi–Liverpool–Wellcome Programme, Blantyre, 3, Malawi; Institute of Infection, Veterinary & Ecological Sciences, University of Liverpool, Liverpool, L69 7ZB, United Kingdom; Malawi–Liverpool–Wellcome Programme, Blantyre, 3, Malawi; School of Life Sciences and Allied Health Professions, Kamuzu University of Health Sciences Blantyre, Blantyre, 265, Malawi; Malawi–Liverpool–Wellcome Programme, Blantyre, 3, Malawi; Institute of Infection, Veterinary & Ecological Sciences, University of Liverpool, Liverpool, L69 7ZB, United Kingdom

**Keywords:** transcriptomics, comparative genomics, lineage evolution, gene expression, antibiotic resistance

## Abstract

Invasive non-typhoidal *Salmonella* (iNTS) disease is a serious bloodstream infection that targets immune-compromised individuals, and causes significant mortality in sub-Saharan Africa. *Salmonella enterica* serovar Typhimurium ST313 causes the majority of iNTS in Malawi. We performed an intensive comparative genomic analysis of 608 *S*. Typhimurium ST313 isolates dating between 1996 and 2018 from Blantyre, Malawi. We discovered that following the arrival of the well-characterized *S*. Typhimurium ST313 lineage 2 in 1999, two multidrug-resistant variants emerged in Malawi in 2006 and 2008, designated sublineages 2.2 and 2.3, respectively. The majority of *S*. Typhimurium isolates from human bloodstream infections in Malawi now belong to sublineages 2.2 or 2.3. To understand the emergence of the prevalent ST313 sublineage 2.2, we studied two representative strains, D23580 (lineage 2) and D37712 (sublineage 2.2). The chromosome of ST313 lineage 2 and sublineage 2.2 only differed by 29 SNPs/small indels and a 3 kb deletion of a Gifsy-2 prophage region including the *sseI* pseudogene. Lineage 2 and sublineage 2.2 had distinctive plasmid profiles. The transcriptome was investigated in 15 infection-relevant *in vitro* conditions and within macrophages. During growth in physiological conditions that do not usually trigger *S*. Typhimurium SPI2 gene expression, the SPI2 genes of D37712 were transcriptionally active. We identified down-regulation of flagellar genes in D37712 compared with D23580. Following phenotypic confirmation of transcriptomic differences, we discovered that sublineage 2.2 had increased fitness compared with lineage 2 during mixed growth in minimal media. We speculate that this competitive advantage is contributing to the emergence of sublineage 2.2 in Malawi.

## Introduction

Non-typhoidal *Salmonella* (NTS) is a key bacterial pathogen that threatens people across the world. Typhimurium and Enteritidis are the two serovars of *Salmonella enterica* responsible for the highest levels of self-limiting gastrointestinal disease in Europe, the USA, and other high-income countries (Zhang et al. 2003). In the industrialized world, NTS has been associated with intensive food production, animal husbandry, and global distribution systems (Majowicz et al. 2010). The *S*. Typhimurium sequence types responsible for gastroenteritis globally include ST19, ST34, and monophasic 1,4, [5],12: i:- variants (Branchu et al. 2018). The diarrhoeal NTS disease is termed dNTS, and is mainly foodborne, posing a significant burden to public health with ~153 million cases and 57 000 deaths per annum worldwide (Kirk et al. 2015, Chirwa et al. 2023).

In contrast, a lethal systemic disease called invasive non-typhoidal *Salmonella* (iNTS) disease has emerged in recent decades in low- and middle-income countries in sub-Saharan Africa. iNTS targets immunocompromised individuals such as adults with HIV, and children under 5 years of age with malaria, malnutrition or severe anaemia (Feasey et al. 2012). In some countries of sub-Saharan Africa, *Salmonella* causes more cases of community-onset bloodstream infections than any other bacterial pathogen (Marchello et al. 2019). In 2017, 535 000 cases of iNTS disease were estimated worldwide, with ∼80% of cases and 77 000 deaths occurring in sub-Saharan Africa (Stanaway et al. 2019).

Clinically, the treatment of iNTS is complicated by multi-drug resistance (MDR) which limits therapeutic options (Crump et al. 2015). Widespread resistance of iNTS pathogens to first-line drugs such as chloramphenicol, ampicillin, and cotrimoxazole has been seen in many countries (Kariuki et al. 2006). This MDR phenotype may be one of the reasons the case fatality rate associated with iNTS is amongst the highest in comparison to any infectious disease (15%) (Marchello et al. 2022). Resistance to second-line drugs such as ceftriaxone, ciprofloxacin, and azithromycin has been reported in a few African countries (Tack et al. 2020). Clearly, the challenge posed by MDR *Salmonella* must be addressed urgently (Gilchrist and MacLennan 2019).

The African iNTS epidemic is mainly caused by two *Salmonella* pathovariants, *S*. Typhimurium sequence type 313 (ST313) and specific clades of *S*. Enteritidis (Kingsley et al. 2009, Okoro et al. 2012, 2015, Feasey et al. 2016). *S*. Typhimurium ST313 is responsible for about two-thirds of clinical iNTS cases that have been reported in Africa (Gilchrist and MacLennan 2019).

It is not certain how these pathogens are transmitted, but there is increasing evidence from case-control studies that ST313 strains are human-associated but not animal-associated within households (Post et al. 2019, Koolman et al. 2022). A recent summary concludes that the available data are consistent with iNTS disease being transmitted person-to-person (Chirwa et al. 2023). Global efforts to combat iNTS infections are currently focused on vaccine development, which has now progressed to Phase 1 clinical trials (Piccini and Montomoli 2020, Skidmore et al. 2023).

Since 1998, continuous sentinel surveillance for fever and bloodstream infections among adults and children has been undertaken at Queen Elizabeth Central Hospital (QECH). This tertiary referral hospital in Blantyre, Malawi, serves an urban population of ∼920 000 with a high incidence of malaria, HIV, and malnutrition (Musicha et al. 2017). Following blood culture of samples collected from patients of all ages presenting with fever, whole genome sequencing identified the ST313 variant of *S*. Typhimurium (Kingsley et al. 2009). Phylogenetic analysis revealed that the chloramphenicol-sensitive ST313 lineage 1 was clonally replaced in Malawi by the chloramphenicol-resistant lineage 2 (Okoro et al. 2012). More recently, an ST313 sublineage II.1 (2.1) emerged from lineage 2 in the Democratic Republic of Congo (DRC) in Central Africa. Sublineage 2.1 had altered phenotypic properties including biofilm formation and metabolic capacity and resistance to azithromycin (Van Puyvelde et al. 2019). An elegant genomic analysis that provides insight into the diversity of *S*. Typhimurium ST19 clades in the context of ST313 lineage 2 clades is also available (Van Puyvelde et al. 2023).

The initial suggestion that ST313 lineage 2 was undergoing evolutionary change in East Africa came from a small study that identified several *S*. Typhimurium ST313 Malawian isolates, dated between 2006 and 2008, that differed from lineage 2 by 22 core-genome single nucleotide polymorphisms (SNPs) (Msefula et al. 2012).

To examine the evolutionary trajectory of *S*. Typhimurium in Malawi at a large scale, we conducted a comparative genomic analysis study focused on 680 isolates dating between 1996 and 2018 (Pulford et al. 2021). We previously reported that ST313 lineage 1 (L1) was replaced by lineage 2 (here designated L2.0), and discovered an antibiotic-sensitive lineage 3 (L3) that emerged in 2016 (Pulford et al. 2021). We have now performed a more intensive phylogenetic analysis of the same collection of *S*. Typhimurium ST313 isolates, most of which caused bloodstream infections in Malawi over two decades. We discovered two novel sublineages named 2.2 (L2.2) and 2.3 (L2.3) that emerged in 2006–2008, and have been replacing L2.0.

Here we present a comprehensive comparative genomic analysis of the most prevalent ST313 L2.2 sublineage and report the results of a functional genomic approach that identified key phenotypic characteristics that distinguish L2.2 from L2.0.

## Materials and methods

### Bacterial strains

**Figure 1.**
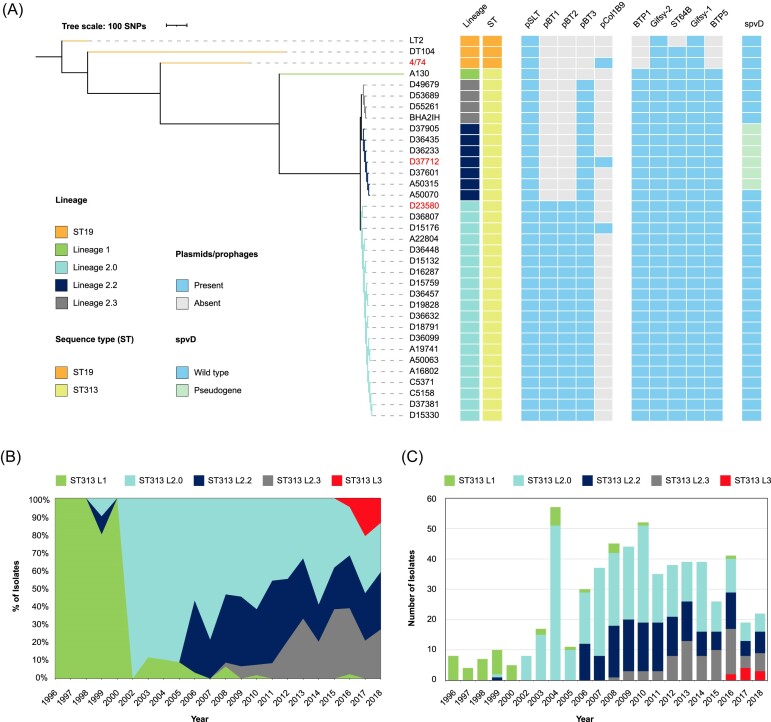
Emergence of *S*. Typhimurium ST313 sublineages L2.2 and L2.3 in Malawi. (A) Evolutionary dynamics of *S*. Typhimurium lineages in Blantyre, Malawi from 1996 to 2018. A maximum likelihood tree constructed with 1000 bootstraps using the GTRGAMMA model in RaxML rooted on ST19, LT2. The genomes of 549 *S*. Typhimurium ST313 isolates from bacteraemic patients at the Queen Elizabeth Hospital in Blantyre, Malawi were used for this analysis. The proportions of the five lineages/sublineages are shown. (B) The total number of isolates of each lineage/sublineage per year. (C) Phylogenetic comparison between representative strains of *S*. Typhimurium ST19 and four ST313 lineages/sublineages (L1, L2.0, L2.2, and L2.3) showing the presence and absence of plasmids, prophages and the *spvD* pseudogene. The complete phylogenetic analysis of 707 *S*. Typhimurium genomes is shown in Fig. S1.

To investigate the evolutionary dynamics of *S*. Typhimurium ST313 L2 in Malawi over a 22-year period, we focused on the large collection of 8 000 *S*. Typhimurium isolates derived from bloodstream infection in hospitalized patients at the QECH, Blantyre, Malawi (Feasey et al. 2015). The collection was assembled by the Malawi–Liverpool–Wellcome Trust Clinical Research Programme (MLW) between 1996 and 2018; the precise annual numbers of isolates are shown in Fig. 1C. A random sub-sampling strategy was used to select 608 isolates for whole-genome sequencing, which included 549 *S*. Typhimurium ST313 isolates (Pulford et al. 2021).

The two *S*. Typhimurium ST313 strains that are the focus of this study are D23580 and D37712. D23580 was isolated from a Malawian 26-month-old child with malaria and anaemia in 2004. D37712 was isolated from the blood of an HIV-positive Malawian male child in 2006. These two African *Salmonella* strains have been deposited in the National Collection of Type Cultures (NCTC). The D23580 (lineage 2.0) strain is available as NCTC 14677. The ST313 sublineage 2.2 strain D37712 is available as NCTC 14678. All bacterial strains are detailed in Table S8.

### Genome sequencing

The assembled genome and annotation of D23580 (Canals et al. 2019b, Kingsley et al. 2009) (L2.0) was obtained from the European Nucleotide Archive (ENA) repository (EMBL-EBI) under accession PRJEB28511 (https://www.ebi.ac.uk/ena/data/view/PRJEB28511). For genome sequencing of D37712 (L2.2), DNA was extracted using the Bioline mini kit, and quality was assessed using gel electrophoresis (0.5% agarose gel, at 30 volts for 18 h). The genome was generated by a combination of long-read sequencing with a PacBio RS II and short-read sequencing on an Illumina HiSeq machine at the Center for Genome Research, University of Liverpool, United Kingdom.

Sequence reads were quality-checked using FastQC version 0.11.9 (Andrews 2010) and MultiQC version 1.8 (Ewels et al. 2016), trimmed using Trimmomatic (Bolger et al. 2014). Hybrid assembly of the Illumina and PacBio sequence reads was done with Unicycler v0.4.7 (Wick et al. 2017).

The assembled genome of *S*. Typhimurium SDT313 L2.2 strain D37712 was deposited in Genbank (GCA_014250335.1, assembly ASM1425033v1). Raw sequencing reads were deposited for both PacBio and Illumina, under BioProject ID PRJNA656698. Sequence Read Archive (SRA) database IDs are SRR12444880 for Illumina and SRR12444881 for PacBio.

### Comparative genomic analyses

To generate the data summarized in Fig. 1C, sequencing data of 29 *S*. Typhimurium ST313 strains (Msefula et al. 2012) were downloaded from EMBL-EBI database (https://www.ebi.ac.uk, accession number ERA015722). Sequence reads were assembled using Unicycler v0.4.8 (Wick et al. 2017). The quality of the assemblies was assessed by Quast v5.0.2 (Gurevich et al. 2013). The N50 value of all assemblies was >20 kb, and the number of contigs was <600.

To construct the phylogenetic tree (Fig. 1C), *Salmonella* Typhimurium strains D23580, D37712, LT2 (GCA_000006945.2), DT104 (GCA_000493675.1), 4/74 (GCA_000188735.1), and A130 (GCA_902500285.1) were added as contextual genomes. Roary was used to make the core gene alignment, construct the gene presence/absence matrix and identify orthologous genes (Page et al. 2015). Phylogenetic trees were constructed using Randomized Accelerated Maximum Likelihood (RAxML) (Stamatakis et al. 2005), and were visualized with the interactive Tree of Life (iToL) online tool (Letunic and Bork 2006).

The assembled genome and annotation of *S*. Typhimurium ST19 representative strain 4/74 (Richardson et al. 2011) were obtained from GenBank (Accession number GCF_000188735.1), while the raw sequencing data of 27 *S*. Typhimurium ST313 strains described in a previous study (Msefula et al. 2012) were downloaded from the EMBL-EBI database (https://www.ebi.ac.uk, accession number ERA015722). The raw reads were assembled using Unicycler v0.4.8 (Wick et al. 2017). The quality of the assemblies was assessed by Quast v5.0.2 (Gurevich et al. 2013). The N50 value of all assemblies was >20 kb, and the number of contigs was <600.

To identify SNPs, Snippy v4.4.0 (https://github.com/tseemann/snippy) was used to map the raw reads against the 4/74 genome. To detect pseudogene-associated SNPs/indels in each sub-lineage, the SNPs/indels that caused nonsense or frameshifted mutations were filtered. The identifications and names of the disrupted genes were summarized, then the wild-type gene sequences were extracted from the 4/74 genome. To validate the pseudogene-associated SNPs/indels, the wild-type gene sequences were used to make a BLAST database with BLAST 2.9.0+ (Camacho et al. 2009). The 29 genome assemblies were queried against the databases, using the BLASTn algorithm to confirm the nonsense and frameshifted mutations in all isolates.

### Phylogenetic analysis of African *Salmonella* Typhimurium isolates dating from 1966 to 2018

To examine the overall population structure of *Salmonella* Typhimurium responsible for blood infection in Malawi (Fig. 1A,B and Fig. S1), the raw reads of 707 published genome sequences were downloaded (Table S7). Trimmomatic v0.36 (Bolger et al. 2014) was used to trim adapters and Seqtk v1.2-r94 (https://github.com/lh3/seqtk) was used to trim low-quality regions using the trimfq flag. Fastqc v0.11.5 (https://www.bioinformatics.babraham.ac.uk/projects/fastqc/) and multiqc v1.0 (http://multiqc.info) were used to pass sequence reads according to the following criteria: passed basic quality statistics, per base sequence quality, per base N content, adapter content and an average GC content of between 47% and 57%. Only high-quality reads were used in the downstream analysis. Sequence reads were aligned to the *S*. Typhimurium D23580 genome using Snippy v4.4.0 with parameter ‘-—mincov 5’. The recombination sites of the alignment were removed by Gubbins (Croucher et al. 2015), and the phylogenetic tree was built with Raxml-ng (Kozlov et al. 2019) using GTR_G models ad 100 bootstraps. The tree was rooted on *Salmonella* Typhi strain CT18 (GCA_000195995.1) as the outgroup. The tree was visualized with the iToL online tool (Letunic and Bork 2006). The sub-lineages were identified with rHierBAPS (Tonkin-Hill et al. 2018). The stacked-area chart and the bar chart showing the percentage and number of isolates from each sub-lineage were made in MS Excel.

### RNA purification and growth conditions

Initially, a screen of transcriptomic gene expression was performed without biological replicates. Total RNA was purified using TRIzol from *S*. Typhimurium D37712 grown in 15 different conditions as described previously (Kröger et al. 2013). To generate statistically robust gene expression profiles, total RNA was subsequently purified using TRIzol from *S*. Typhimurium D37712 grown in four *in vitro* growth conditions (ESP, anaerobic growth, NonSPI2, and InSPI2) with three biological replicates as described previously (Kröger et al. 2013). RNA was isolated from intra-macrophage D37712 following infection of RAW264.7 murine macrophages using our published protocol (Srikumar et al. 2015).

### RNA-seq of *S*. Typhimurium strain D37712 using Illumina technology

For transcriptomic analyses, cDNA samples were prepared from *S*. Typhimurium RNA by Vertis Biotechnologie AG (Freising, Germany). RNA was first treated with DNase and purified using the Agencourt RNAClean XP kit (Beckman Coulter Genomics). RNA samples were sheared using ultrasound, treated with antarctic phosphatase and re-phosphorylated with T4 polynucleotide kinase. RNA fragments were poly(A)-tailed using poly(A) polymerase and an RNA adapter was ligated to the 5′-phosphate of the RNA. First-strand cDNA synthesis was performed using an oligo(dT)-adapter primer and M-MLV reverse transcriptase. The resulting cDNA was PCR-amplified to ∼10–20 ng/µl. The cDNA was purified using the Agencourt AMPure XP kit. The cDNA samples were pooled using equimolar amounts and size fractionated in the size range of 200–500 bp using preparative agarose gels. The cDNA pool was sequenced on an Illumina NextSeq 500 system using a 75 bp read length.

For the biological replicates of the four growth conditions (ESP, anaerobic growth (abbreviated as NoO2), NonSPI2, and InSPI2) and the intra-macrophage RNA, cDNA samples were generated as above with some improvements in library preparation. First, after fragmentation with ultrasound, an oligonucleotide adapter was ligated to the 3′ end of the RNA molecules. Second, first-strand cDNA synthesis was performed using M-MLV reverse transcriptase and the 3′ adapter as primer, and, after purification, the 5′ Illumina TruSeq sequencing adapter was ligated to the 3′ end of the antisense cDNA. Sequencing of the cDNA was performed as described above. All raw sequencing reads were deposited to the Gene Expression Omnibus (GEO) database under accession GSE161403.

### RNA-seq and dRNA-seq read processing and visualization

RNA-seq data from *S*. Typhimurium 4/74 and D23580 were extracted from previously published experiments (Canals et al. 2019b, Kröger et al. 2013, Srikumar et al. 2015; GEO dataset GSE119724). A combined reference genome was generated that contained the D23580 chromosome plus plasmids pBT1, pBT2, pBT3, pSLT-BT (from D23580) and the D37712 plasmid pCol1B9^D37712^. All reads were aligned and quantified using Bacpipe v0.8a (https://github.com/apredeus/multi-bacpipe). Briefly, basic read quality control was performed with FastQC v0.11.8. RNA-seq reads were aligned to the genome sequence using STAR v2.6.0c using ‘–alignIntronMin 20 –alignIntronMax 19 –outFilterMultimapNmax 20’ options. A combined GFF file was generated by Bacpipe, where all features of interest were listed as a ‘gene’, with each gene identified by a D37712 locus tag. Subsequently, read counting was done by featureCounts v1.6.4, using options ‘-O -M –fraction -t gene -g ID -s 1’. For visualization, scaled gedGraph files were generated using bedtools genomecov with a scaling coefficient of 10^9^/(number of aligned bases), separately for sense and antisense DNA strands. Bedgraph files were converted to bigWig using bedGraphToBigWig utility (http://hgdownload.soe.ucsc.edu/admin/exe/linux.x86_64/). Coverage tracks, annotation, and genome sequence were visualized using JBrowse v1.16.6. TPM were calculated for all samples and used as absolute expression values (Table S5). A conservative cut-off was used to distinguish between expressed (TPM > 10) and not expressed (TPM ≤ 10), as we previously described (Kröger et al. 2013). Relative expression values were calculated by dividing the TPM value for one condition in one strain by the TPM value for the same condition in a different strain. Before the calculation, all TPM values below 10 were set up to 10. A conservative fold-change cut-off of 3 was used to highlight differences in expression between strains.

### Differential gene expression analysis with multiple biological replicates

For differential expression analysis of *S*. Typhimurium strains 4/74, D23580, and D37712, the raw counts (Table S4) from 3 to 5 biological replicates in four growth conditions were used (ESP, anaerobic growth (abbreviated as NoO2), NonSPI2, and InSPI2). Differential expression analysis was done using DESeq2 v1.24.0 with default settings. A gene was considered to be differentially expressed if the absolute value of its log2 fold change was at least 1 (i.e. fold change > 2), and the adjusted *P* value was <.001.

### The SalComD37712 community data resource, and the associated Jbrowse genome browser

SalCom provides a user-friendly Web interface that allows the visualization and comparison of gene expression values across multiple conditions and between strains. Particular genes can be selected through pre-defined lists of interest, such as all sRNAs or all genes belonging to a specific pathogenicity island. The resulting heatmap-style display highlights expression differences and provides access to the rich, manually curated annotation of strains D37712 and D23580. The actual values behind the display can be downloaded for further processing, and a link connects the current view to a genome browser interface.

Visualization of all the RNA-seq and dRNA-seq (TSS) coverage tracks in JBrowse 1.16.6 shows sequence reads mapped against the combined reference genome described above. Overall, the genomic distance between strains 4/74 and D23580 (~1000 SNPs, or ∼1 SNP per 5000 nucleotides), and between D37712 and D23580 (~30 SNPs, ∼1 SNP per 150 000 nucleotides) allowed the alignment of RNA-seq reads to the simplified combined reference genome without significant loss of reads. The combined reference genome facilitated a direct comparison of gene coverage as well as TSS. The unified browser is hosted at http://hintonlab.com/jbrowse/index.html?data=Combo_D37/data.

### Phenotypic and mixed competitive growth experiments

The swimming motility of *S*. Typhimurium strains D37712, D23580 and 4/74 was determined by a plate assay (Canals et al. 2019b), which involved spotting 3 µl overnight culture onto 0.3% LB agar. Relative motility of the three strains was assessed by migration diameter after 4 h and 8 h of incubation at 37°C.

Relative expression of the *ssaG* SPI2 promoter in strains D23580 and D37712 was measured at the single cell level via GFP fluorescence. Following the construction of a kanamycin-sensitive derivative of D23580 (strain JH4235), a P*ssaG::gfp*^+^ transcriptional fusion was incorporated into the chromosome of JH4235 and D37712 by inserting the *gfp^+^* gene downstream of the *ssaG* gene, under the control of the P*ssaG* promoter. The P*ssaG::gfp*^+^ D23580 derivative (JH4692), and the P*ssaG::gfp*^+^ D37712 derivative (JH4693) are listed in Table S8.

The strains JH4692 and JH4693 were genome sequenced to confirm the integrity of the transcriptional fusions, and to verify that unintended nucleotide changes had not arisen. Following growth in 25 ml non-inducing NonSPI2 media in a 250 ml flask at 37°C with shaking at 220 rpm for ~8 h until OD_600_ = 0.3, fluorescence was determined with a BD FACSAria Flow Cytometer. The relative fluorescence of the two strains JH4692 and JH4693, and the numbers of individual fluorescent bacteria that expressed the P*ssaG::gfp*^+^ promoter, were determined with FlowJo VX software.

The relative fitness of *S*. Typhimurium strains D37712 and D23580 was assessed in two independent mixed-growth experiments. First, kanamycin-resistant derivatives of each strain were constructed by inserting the *aph* kanamycin resistance gene into the chromosome at the intergenic region between the *STM4196* and *STM4197* genes, a region that we have previously shown to be transcriptionally silent (Canals et al. 2019b). The strains were designated D23580::Km^R^ JH3794 and D37712::Km^R^, JH4232. Mixed cultures of wild-type or kanamycin-resistant derivatives of each strain were grown overnight in LB, InSPI2, and NonSPI2 media in a 250 ml flask at 37°C with shaking at 220 rpm. Following plating on LB agar or LB + kanamycin, colonies were counted and the ratio of bacterial strains was determined. To confirm that the insertion of kanamycin resistance at the intergenic region between *STM4196* and *STM4197* did not impact upon fitness, a mixed-growth experiment was done in both LB and NonSPI2 media (Fig. S7).

Second, to independently assess relative fitness, Tn*7*-based plasmids (Schlechter and Remus-Emsermann 2019) were used to construct chromosomal sGFP2 and mScarlet derivatives of *S*. Typhimurium strains D23580 (sGFP2 derivative: JH4694; mScarlet derivative: JH4695) and D37712 (sGFP2 derivative: JH4696; mScarlet derivative: JH4697). The gene cassettes were inserted into the *S*. TyphimuriumTn*7* insertion site between the gene *STMMW_38 451* and *glmS*. Mixed cultures of pairs of fluorescently labelled strains were grown in NonSPI2 media at 37°C with shaking at 220 rpm for ~8 h until OD_600_ = 0.3. Levels of green and red fluorescence were determined with a BD FACSAria Flow Cytometer.

## Results

### Identification of *S*. Typhimurium ST313 sublineages 2.2 and 2.3 in Malawi

The emergence of the ST313 lineage 2 genotype in Malawi in 2002 prompted us to hypothesize that subsequent evolution would select variants with increased fitness, leading to the clonal expansion of one or more sublineages by outcompeting previously dominant genotypes. We investigated this hypothesis by conducting a detailed core-gene SNP-based maximum likelihood (ML) phylogenetic analysis to investigate the population structure of *S*. Typhimurium ST313 L2.0 (Fig. S1). As well as identifying members of the antibiotic-sensitive lineage 3, reported previously (Pulford et al. 2021), we discovered that ST313 L2 comprised three phylogenetically distinct sublineages that differed by a total of 39 SNPs. The *S*. Typhimurium ST313 reference strain D23580 (Kingsley et al. 2009) belongs to the ST313 L2.0 lineage (Fig. 1A). As ST313 sublineage L2.1 had been defined previously (Van Puyvelde et al. 2019), the new sublineages which belonged to different hierBAPS level 2 clusters were designated L2.2 and L2.3 (Fig. 1A and Fig. S1). In total, we identified 151 L2.2 isolates, 74 L2.3 isolates, and 350 L2.0 isolates.

In Blantyre, Malawi, *S*. Typhimurium ST313 L2.2 was first detected in 2006, and L2.3 was initially observed in 2008 (Fig. 1B,C). Both L2.2 and L2.3 increased in prevalence at the QECH in Blantyre in subsequent years. By 2018, L2.2 and L2.3 had largely replaced L2.0 (Fig. 1B,C). Our published Bayesian (Bayesian Evolutionary Analysis by Sampling Trees (BEAST)) analysis (Pulford et al. 2021) estimated that the Most Recent Common Ancestor (MRCA) of ST313 lineage 2 dates back to 1948 (95% HPD (Highest Posterior Density) = 1929–1959).

To understand the accessory gene complement of L2.2 and L2.3, we compared the genomes of seven L2.2 isolates and four L2.3 isolates with 17 L2.0 isolates, ST313 L1 and ST19 and the results are shown in Fig. 1A and Table S1. *S*. Typhimurium strain D23580 is the representative strain of L2.0 (Kingsley et al. 2009), for which we previously used long-read sequencing and other approaches to thoroughly characterize the chromosomal and plasmid complement (Canals et al. 2019b).

### Antimicrobial resistance

**Figure 2.**
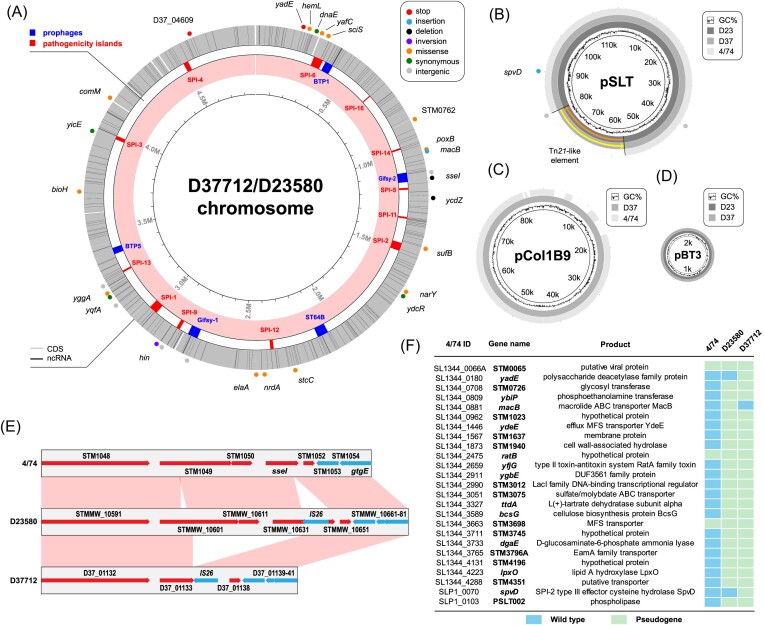
Key genetic similarities and differences between the chromosome and plasmid profiles of D23580 (lineage 2) and D37712 (L2.2). (A) A comparison of the D23580 (L2.0) and D37712 (L2.2) chromosomes. The dots around the chromosome are different kinds of SNPs identified. Phages and *Salmonella* pathogenicity islands are shown in blue and red respectively. (B) Plasmid profile of D37712 versus D23580. The pSLT-BT virulence plasmid is present in both D37712 and D23580 and carries the Tn-21 transposable element; (C) pCol1B9 is present in D37712 and absent from D23580; and (D) pBT3 is present in both D37712 and D23580. (E) Absence of sseI gene and the STM1050 coding sequence in L2.2 (D37712), as compared to *S*. Typhimurium ST19 4/74 and *S*. Typhimurium ST313 L2.0 (D23580). (F) List of pseudogenes in D37712 and D23580, with reference to 4/74. The colour blue means pseudogene/disrupted gene while grey indicates functional genes. macB is a pseudogene in D23580 (L2.0) but not in L2.2, while spvD is a pseudogene in L2.2 but not in L2.0. All L2.2 strains share similar pseudogenes.

MDR variants of *S*. Typhimurium with resistance to ampicillin and cotrimoxazole were detected at an early stage of the iNTS epidemic, from 1997 onward (Gordon et al. 2008). Multidrug-resistant variants of *S*. Typhimurium ST313 that were no longer susceptible to chloramphenicol, ampicillin and cotrimoxazole subsequently emerged in Malawi (Gordon et al. 2008) and have been reported elsewhere in sub-Saharan Africa by the Global Enteric Multicenter Study (GEMS) study (Kasumba et al. 2021). The *S*. Typhimurium ST313 L2.0, L2.2, and L2.3 isolates shared the same MDR profiles (resistance to chloramphenicol, ampicillin, and cotrimoxazole), and carried identical IS21-associated antimicrobial gene cassettes within the pSLT-BT plasmid (Fig. 2B).

### Comparative genomics of *S*. Typhimurium ST313 sublineage 2.2

Because *S*. Typhimurium ST313 L2.2 was the predominant novel sublineage in Blantyre, Malawi in 2018, we focused on L2.2 for the remainder of this study. We used the phylogeny (Fig. 1A) to select strain D37712 as a representative of L2.2. D37712 was isolated from the blood of an HIV-positive Malawian male child and has been deposited in the National Collection of Type Cultures as NCTC14678. The draft genome sequence of D37712 was obtained in 2012 with Illumina technology, an assembly that comprised 27 individual contigs (Msefula et al. 2012). To generate a reference-quality genome, we resequenced D37712 with both long-read PacBio and Illumina short-read technologies. Our hybrid strategy generated a complete genome assembly that included one circular chromosome and three plasmids (see Materials & Methods; GenBank CP060165, CP060166, CP060167, and CP060168). This high-quality genome sequence allowed us to conduct a detailed comparison between the genomes of L2.2 strain D37712 and L2.0 strain D23580 (accession number FN424405), summarized in Fig. 2 and Table S2.

Overall, the gene content of the two strains was largely equivalent. The D23580 annotation contains 4823 protein-coding and pseudogenes and 287 small RNA (sRNA) genes that we identified previously (Canals et al. 2019b), while D37712 contains 4821 protein-coding and pseudogenes and the same 287 sRNAs. In total, the D37712 and D23580 genomes shared 4729 orthologous protein-coding genes and pseudogenes. The 104 protein genes that differ are encoded by the pSLT ^D37712^, pBT1^D37712^, and pCol1B9^D37712^ plasmids.

### Overview of D23580 and D37712 genomes

The chromosomes of D23580 and D37712 are 4 879 402 and 4 876 060 bp, respectively, about the same size as other *S*. Typhimurium genomes (Kingsley et al. 2009, Branchu et al. 2018). The D23580 and D37712 strains share an identical prophage profile, with both strains carrying five prophages (BTP1, Gifsy-2, ST64B, Gifsy-1, and BTP5) located at the same positions on the chromosome (Fig. 2A) (Owen et al. 2017).

### Comparison of D23580 and D37712 chromosomes

The detailed genomic comparison of D37712 with D23580 showed that the sizes of the two chromosomes varied by only 3342 bp. Overall, the only differences between the genomes of the L2.0 and L2.2 strains were 26 chromosomal SNPs and small indels, plus one large deletion, and an inversion of the *hin* switch. In-depth annotation of the nucleotide variants identified three putative loss-of-function mutations (two stop mutations, one frameshift insertion), one disruptive in-frame deletion, four synonymous mutations, 13 missense mutations, and five intergenic variants, summarized in Fig. 2A. None of the SNP differences that distinguished D37712 from D23580 were located within 150 nucleotides of a Transcriptional Start Site (TSS) (Canals et al. 2019b), and so would not be predicted to modulate gene expression.

The 3358 bp-long deletion of a Gifsy-2 prophage-associated region that spanned the *sseI* pseudogene of D23580 (STMMW_10 631) removed two coding sequences (STM1050-51; STMMW_10611-STMMW_10 631), and substantially truncated the STM1049 (STMMW_10 601) gene (Fig. 2E). The *sseI* gene encodes a cysteine hydrolase effector protein that modulates the directional migration of dendritic cells during systemic infection (Brink et al. 2018). In strain D23580, the insertion of an IS26 transposable element inactivated the *sseI* gene (Kingsley et al. 2009), causing increased dendritic cell-mediated dissemination of strain D23580 during infection (Carden et al. 2017). We used an independent Polymerase Chain Reaction (PCR)-based approach to confirm that the 3358 bp deletion had removed the *sseI* gene from the chromosome of strain D37712 (Fig. S2).

### Comparison of D23580 and D37712 plasmids

Here we put the genetic features of the representative strains for ST313 L2.0 and L2.2 into context with other isolates belonging to the Lineage 2 sublineages. ST313 L2.0 strain D23580 carries four plasmids, pSLT-BT, pBT1, pBT2, and pBT3 (Kingsley et al. 2009). In contrast, ST313 L2.2 has a distinct plasmid complement (Figs. 1A and 2B,C,D). The plasmid profiles of D23580 and D37712 were confirmed by a combination of Illumina (short-read) and PacBio (long-read) sequencing (Materials and Methods).

In summary, strain D37712 carried the pSLT-BT, pBT2, and pCol1B9 plasmids as detailed below. Both D23580 and D37712 strains carried a variant of the pSLT-BT virulence plasmid (Kingsley et al. 2009) that contains a Tn21-like transposable element with five antibiotic resistance genes. The D37712 version of pSLT-BT only differs from the pSLT-BT of D23580 in two important t ways (Fig. 2B). Firstly, the Tn21-like element is inserted in the opposite direction with regards to the rest of the plasmid, suggesting that the transposable element remains active. Secondly, three nucleotide variants were identified in the pSLT-BT carried by D37712, two deletions in noncoding regions, and one frameshift insertion that generates a pseudogene of *spvD*.

Plasmid pCol1B9 was of particular interest because it was absent from D23580, but was present in *S*. Typhimurium ST19 strain 4/74 (Richardson et al. 2011, Fig. 1A). 4/74 is the parent of *S*. Typhimurium SL1344, a strain that has been used extensively for the study of *S*. Typhimurium pathogenesis and gene regulation in recent decades (Rankin and Taylor 1966, Kröger et al. 2012). Our new annotation of the pCol1B9-like plasmid identified 95 distinct protein-coding genes, while the previously published annotation of pCol1B9^4/74^ assigned 101 protein-coding genes. Some of these represent annotation discrepancies, while others represent true genetic differences (Fig. S3).

Following careful examination, we identified 14 genes unique to pCol1B9^D37712^, and 20 genes unique to pCol1B9^4/74^. There were 81 genes carried by both plasmids. Interestingly, pCol1B9^D37712^ lacked the colicin toxin–antitoxin system that both gave pCol1B9 its name, and provides *Salmonella* with a competitive advantage in the gut (Nedialkova et al. 2014). The pCol1B9^D37712^ plasmid carried a locus that was absent from pCol1B9^4/74^, namely the *impC-umuCD* operon (Fig. S3) which encodes the error-prone DNA polymerase V responsible for the increased mutation rate linked to the SOS stress response in *E. coli* (Sikand et al. 2021).

### Comparison of pseudogene status of D23580 and D37712

Our comparative genomic analysis focused on the pseudogenes found in strains 4/74, D23580, and D37712 (Fig. 2F and Table S3). The pseudogenization of several D23580 genes, compared with strain 4/74, has been linked to the invasive phenotype of African *Salmonella* ST313 (Kingsley et al. 2009). We found that the pseudogene complement of D23580 was largely conserved in D37712, consistent with inheritance from a common ancestor. We have recently reported the role of the MacAB-TolC macrolide efflux pump in the virulence of *S*. Typhimurium ST313 and showed experimentally that *macB* was an inactive pseudogene in D23580 (Honeycutt et al. 2020). Interestingly, the *macB* gene is functional in D37712. Compared with D23580, three additional D37712 genes were pseudogenized (*spvD, yadE*, and STMMW_42 692, as detailed in Table S3). YadE is a predicted polysaccharide deacetylase lipoprotein. The functional impact of these pseudogenes on L2.2 remains to be established.

Overall the chromosomes of ST313 lineage 2 and sublineage 2.2 were highly conserved and differed by just 29 SNPs/small indels, and a 3-kb deletion in the Gifsy-2 prophage region. The ST313 lineage 2 and sublineage 2.2 have distinct plasmid profiles.

### Transcriptional landscape of *S. Typhimurium* ST313 sublineage L2.2

Previously, we characterized the primary transcriptome of two other *S*. Typhimurium strains, 4/74 and D23580, using a combination of multi-condition RNA-seq and differential RNA-seq (dRNA-seq) techniques (Canals et al. 2019b, Kröger et al. 2013). To identify the TSS of strain D37712, we analysed a pooled sample containing RNA from 15 *in vitro* conditions by dRNA-seq and RNA-seq as detailed previously (Kröger et al. 2013). The high similarity between the D23580 and D37712 chromosomes allowed us to map the curated set of TSS that were previously defined for D23580 (Hammarlöf et al. 2018) onto a combined D37712/D23580 reference genome. To allow individual TSS to be examined in particular chromosomal or plasmid regions, data from both the dRNA-seq and pooled RNA-seq experiments can be visualized in our online genome browser (http://hintonlab.com/jbrowse/index.html?data=Combo_D37/data).

### Preliminary gene expression profiling of *S*. Typhimurium ST313 sublineage L2.2

Given the high level of similarity between the genomes of L2.2 and L2.0, we went on to identify differences at the transcriptional level. We performed a multi-condition RNA-seq-based transcriptomic analysis of gene expression profiles of L2.2 strain D37712 without biological replicates.

**Figure 3.**
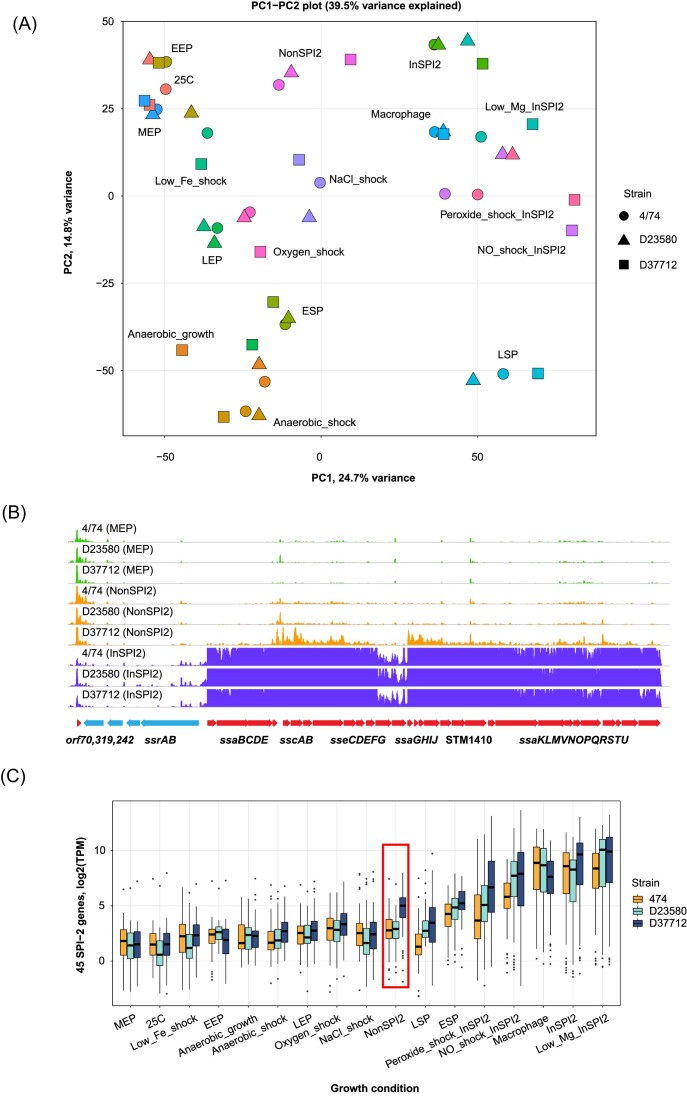
General comparison of expression profiles of strains 4/74, D23580, and D37712 under 17 different *in vitro* conditions. (A) Principal component analysis (PCA) plot of the individual RNA-seq samples, indicating the overall similarity in gene expression between the three strains. The 17 growth conditions have been defined previously (Kröger et al. 2013). (B) Visualization of SPI-2 pathogenicity island expression with the Jbrowse genomic browser, at mid-exponential phase (MEP), InSPI2, and NonSPI2 *in vitro* conditions, which can be accessed here. (C) Boxplot visualization of SPI-2 gene expression at mid-exponential phase (MEP), InSPI2, and NonSPI2 *in vitro* conditions. The y-axis shows the combined log TPM values for 45 genes located in the SPI2 pathogenicity island, namely *ssaU, ssaT, ssaS, ssaR, ssaQ, ssaP, ssaO, ssaN, ssaV, ssaM, ssaL, ssaK, STnc1220, STM1410, ssaJ, ssaI, ssaH, ssaG, sseG, sseF, sscB, sseE, sseD, sseC, sscA, sseB, sseA, ssaE, ssaD, ssaC, ssaB, ssrA, ssrB, orf242, orf319, orf70, ttrR, ttrS, ttrC, ttrB, ttrA, orf408, orf245, orf32, and orf48*. The elevated expression of SPI-2 genes in strain D37712 cultured in NonSPI2 media is highlighted in a red box.

This comparative transcriptomic screen was based on our published approach (Canals et al. 2019b). Specifically, we used 15 individual infection-relevant *in vitro* conditions (Kröger et al. 2013) and did intra-macrophage transcriptome profiling using the protocol previously established for *S*. Typhimurium ST19 (Srikumar et al. 2015). The RNA-seq samples were mapped to a combined reference genome, which included the annotated D23580 chromosome (Canals et al. 2019b), as well as all the plasmids described earlier (pSLT-BT, pBT1, pBT3, and pCol1B9; see Methods). The initial RNA-seq assessment (detailed in Methods) involved 2–4 M non-rRNA/tRNA reads per sample, allowing gene signatures specific for each *in vitro* condition to be identified. Although single-replicate RNA-seq experiments of this type cannot be used for statistically robust differential gene expression analysis, they do provide a useful screening approach for identifying growth conditions to be used for follow-up experiments. The individual RNA-seq experiments showed broad condition-specific similarities in gene expression between strains 4/74, D37712, and D23580 (Fig. 3A). The gene expression values from each profiled condition are available as raw counts and Transcripts Per Million (TPMs) in Tables S4 and S5.

To select the ideal environmental conditions to use for subsequent experiments, we assessed the expression profiles of known *Salmonella* pathogenicity islands which were broadly similar in strains D37712, and D23580. Although the expression profile of the SPI2 pathogenicity island was broadly similar between D37712, D23580 and 4/74 in most growth conditions, the SPI2 genes of D37712 were highly up-regulated in a single growth condition, NonSPI2 (Fig. 3B,C). NonSPI2 is a minimal medium with a neutral pH and a relatively high level of phosphate, in which *S*. Typhimurium does not usually express the SPI2 pathogenicity island (Löber et al. 2006, Kröger et al. 2013). This intriguing observation prompted us to perform a more discriminating set of transcriptomic experiments, as described below.

### Differential gene expression analysis of *S*. Typhimurium D37712 versus D23580 in four *in vitro* conditions with multiple biological replicates

To define the transcriptional signature of strain D37712 more accurately, we generated RNA-seq data from D37712 grown in four *in vitro* conditions that stimulate expression of the majority of virulence genes: Early Stationary Phase (ESP), anaerobic growth, NonSPI2, and InSPI2, with multiple (3–4) biological replicates. The combination of acidity (pH 5.8) and low phosphate (0.4 mM Pi) in the InSPI2 media stimulates transcription of SPI2 genes in *S*. Typhimurium (Löber et al. 2006, Kröger et al. 2013). The NonSPI2 condition is based on the same PCN media recipe as InSPI2 media, but is neutral (pH 7.4), and contains higher levels of phosphate (25 mM Pi) (Löber et al. 2006, Kröger et al. 2013).

**Figure 4.**
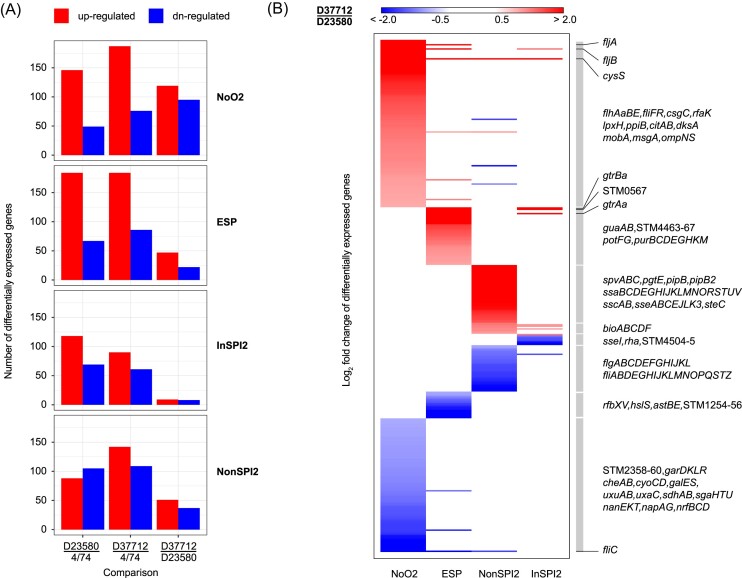
Differential gene expression of *S*. Typhimurium 4/74, D37712, and D23580 under four *in vitro* conditions. (A) Boxplots indicating the number of differentially expressed genes identified in the following *in vitro* growth conditions: early stationary phase, ESP; anaerobic growth, NoO2; SPI-2 inducing medium, InSPI2; SPI-2 non-inducing minimal medium, NonSPI2. Multiple (3–5) biological replicates were used for comparison. DESeq2 was used for differential analysis; only genes with |log2FC| ≥ 1 and with the adjusted *P* value ≤.001 were retained. (B) Heatmap of the genes differentially expressed between D23580 and D37712. Functional groups and operons of interest are highlighted on the right of Panel B.

We compared the results with our published transcriptomic data for *S*. Typhimurium strains 4/74 and D23580 (Canals et al. 2019b, Kröger et al. 2013). Differential expression analysis with DEseq2, with conservative cut-offs (fold change ≥ 2, FDR ≤ 0.001), showed that the gene expression profiles of D37712 and D23580 were broadly similar, and shared key differences to the transcriptional profile of strain 4/74 under each of the four *in vitro* conditions (Fig. 4A). The differential expression results are summarized in Table S6.

We specifically investigated transcription of the *pgtE* gene, which encodes the outer-membrane protease previously linked to the ability of African *Salmonella* ST313 to resist human serum killing (Hammarlöf et al. 2018). Compared to 4/74, the *pgtE* gene of both the D23580 and D37712 strains showed a similar pattern of up-regulation by a factor of seven to 18 across all conditions. This finding is consistent with the fact that D37712 carries the same T nucleotide in the -10 region of the *pgtE* promoter that is responsible for increased expression of the *pgtE* transcript in strain D23580 (Hammarlöf et al. 2018).

There were no statistically significant changes in the expression of the majority (92%) of the 4729 orthologous coding genes shared by D37712 and D23580. We identified a total of 364 genes that were differentially expressed in at least one growth condition between D37712 and D23580 as follows: ESP (69 differentially expressed genes), anaerobic growth (214 differentially expressed genes), NonSPI2 (88 differentially expressed genes), and InSPI2 (17 differentially expressed genes; Fig. 4B).

Overall, the differentially expressed genes that distinguished D37712 from D23580 only showed expression differences in a single growth condition rather than across all conditions. The differentially expressed genes included flagellar genes (down-regulated), SPI2-associated genes(up-regulated), and genes involved in general and anaerobic metabolism (down-regulated).

SPI2 pathogenicity island genes play a key role in the intracellular replication of *S*. Typhimurium, and encode the type III secretion system that is responsible for the translocation of key effector proteins into mammalian cells (Jennings et al. 2017). The RNA-seq data showed that SPI2 genes were expressed at similarly high levels in both D37712 and D23580 strains following induction (InSPI2 media; Fig. 4B), and confirmed that the key SPI2 expression difference was only seen in strain D37712 under non-inducing growth conditions (NonSPI2 media). It is important to put this differential SPI2 expression into context. D37712 expresses SPI2 genes at about a 10-fold higher level than D23580 during growth in non-inducing NonSPI2 media, but the actual level of expression was 20-fold less than the level stimulated by growth in SPI2-inducing conditions (InSPI2 medium).

The up-regulation of *fljA* and *fljB* and the down-regulation of *fliC* in D37712, compared to D23580 in all four growth conditions likely reflects the opposite orientation of the *hin* switch in the D37712 genome compared to D23580. This type of *hin* inversion occurs frequently in *S*. Typhimurium (Johnson and Simon 1985).

Another gene that was up-regulated in D37712 across all profiled conditions was the chromosomally encoded *cysS^chr^*, which encodes cysteine-tRNA synthetase. Previously, we reported that transcription of the *cysS^chr^* of strain D23580 was uniformly down-regulated compared to 4/74. This down-regulation was compensated by the presence of a pBT1 plasmid-encoded cysteine-tRNA synthetase (Canals et al. 2019a). Accordingly, the increased expression of the chromosomal *cysS* gene in D37712 was consistent with the absence of the pBT1 plasmid. Our comparative transcriptomic analysis showed that expression levels of *cysS* were similar in D37712 and 4/74 under all growth conditions.

Numerous virulence genes and operons were differentially expressed between D23580 and D37712. The SPI-16-associated *gtrABCa* operon (STM0557, STM0558, and STM0559) is responsible for adding glucose residues to the O-antigen subunits of LPS that enhance the long-term colonization of the mammalian gastrointestinal tract by *S*. Typhimurium ST19 (Bogomolnaya et al. 2008). We found that the *gtrABCa* genes were significantly up-regulated in several conditions in D37712, compared to both D23580 and 4/74.

The *spvABCD* operon of D37712 was up-regulated under non-SPI2-inducing growth conditions, compared to D23580. A signature pseudogene of ST313 L2.2 is the frameshift insertion in the *spvD* gene that generates a truncated version of the SpvD protein. The H199I mutation at position 199 and the associated 17 amino acid truncation is predicted to ablate the activity of the SpvD cysteine protease (Grabe et al. 2016). SpvD negatively regulates the NF-B signaling pathway and promotes virulence of *S*. Typhimurium in mice. The functional consequences of the *spvD* variant of ST313 L2.2 strain D37712 and the up-regulation of expression of the *spvABCD* operon remain to be established experimentally.

### The SalComD37712 community transcriptional data resource

To allow scientists to gain their own biological insights from the analysis of this rich transcriptomic dataset, the transcriptomic and gene expression data generated in this study are presented online in a new community resource, SalComD37712 (https://tinyurl.com/SalcomD37712). The data resource shows the expression levels of all D37712 coding and non-coding genes, including both chromosomal and plasmid-encoded transcripts. The SalComD37712 website complements our existing SalComD23580 (https://tinyurl.com/SalComD23580) resource, and adds an inter-strain comparison of gene expression profiles between D37712 and D23580 as well as normalized gene expression values (TPM), using an intuitive heat map-based approach. SalComD37712 included our published RNA-seq data (Canals et al. 2019b), re-analysed with an updated bioinformatic pipeline and a combined reference genome (see Methods). This online resource facilitates the intuitive interrogation of transcriptomic data as described previously (Perez-Sepulveda and Hinton 2018).

Additionally, we generated a unified genome-level browser that provides access to the *S*. Typhimurium L2.2 D37712 transcriptome, in the context of our previously published RNA-seq data for the L2.0 strain D23580 and the ST19 strain 4/74. This novel ‘combo’ browser is available at http://hintonlab.com/jbrowse/index.html?data=Combo_D37/data.

### Identification of phenotypes that distinguish ST313 sublineage L2.2 from L2.0

**Figure 5.**
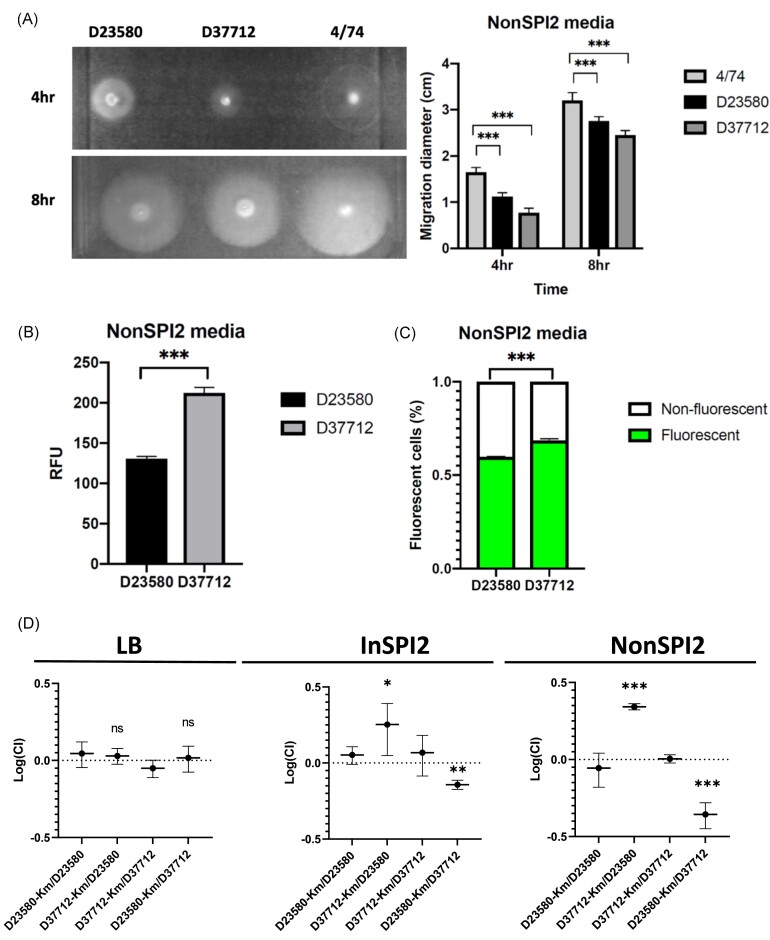
Phenotypes that distinguish ST313 L2.2 from ST313 L2.0. (A) Swimming motility assay of strains D23589, D37712 and 4/74, with a representative plate shown on the left. Average migration diameters were measured after 4 and 8 h. Each bar represents the mean of three biological replicates, with *error bars* showing standard deviation. A significant difference (***) indicates *P* value (*t*-test) < .001. In Panels B & C, comparison of *ssaG* expression by flow cytometry using D23580 and D37712 derivatives containing a chromosomal *ssaG*-GFP^+^ transcriptional fusion, strains SZS008 and SZS032, respectively. Cells were collected at 8 h after inoculation in NonSPI2 media. Ten thousand events were acquired for each sample. (B) Mean fluorescent intensity signal of *ssaG*-GFP^+^ for D23580 (SZS008, dark grey) and D37712 (SZS032, grey) grown in NonSPI2 media. A significant difference (***) indicates *P* value (*t*-test) < .001. (C) The proportions of bacterial cells that expressed *ssaG*-GFP^+^ during growth in NonSPI2 media were determined. The percentage of GFP-expressing (green) and non-fluorescent cells (white) for D23580 (SZS008) and D37712 (SZS032) is shown. Each bar represents the mean of three biological replicates, error bars show standard deviation. A significant difference (***) indicates *P* value (*t*-test) < .001. (D) Relative fitness of wild-type D23580 and D37712 and their kanamycin-resistant derivatives. Bacterial numbers were determined after overnight culture of a 1:1 mixture (wild type versus Km^R^) in LB (left), InSPI2 (middle) and NonSPI2 (right) media. Each dot represents the log-transformed mean competitive index of three biological replicates with *error bars* representing a 95% confidence interval from the standard deviation. A log number higher than 0 reflects the increased fitness of kanamycin-resistant derivatives. *P* values were determined by *t*-test (***: *P* < .001; **: *P* < .01; *: *P* < .05; ns: not significant).

To explore the phenotypic impact of the transcriptomic signature of L2.2 (D37712), we performed a series of motility experiments, fluorescence-based gene expression experiments and mixed-growth assays. D33712 showed a significantly decreased level of motility on NonSPI2 minimal media, compared with both the ST19 strain 4/74 and the L2 D23580 strain (Fig. 5A). This finding was consistent with the transcriptomic data, which showed down-regulation of D37712 flagellar genes compared with D23580 in the NonSPI2 condition (Fig. 4). In contrast, no differential expression of flagellar genes was seen between D33712 and D23580 in the InSPI2 growth condition (Fig. 4). The decreased motility phenotype may be linked to the inversion of the *hin* element detailed above. The flagella system encodes a distinct type III secretion apparatus responsible for the dual functions of bacterial motility and activation of the mammalian innate immune system via TLR5 (Lai et al. 2013).

A key transcriptomic finding for strain D33712 was the expression of SPI2 genes during growth in an unusual environmental condition (NonSPI2) (Figs. 3B,C and 4B). NonSPI2 media differs from InSPI2 media by having a higher pH (pH7.4 versus pH5.8) and a higher level of phosphate (Löber et al. 2006). This apparent differential expression of SPI2 genes at the transcriptomic level under non-inducing conditions led us to investigate the expression of SPI2 at a single-cell level using fluorescence transcriptional fusions. First, we introduced an *ssaG*-GFP^+^ transcriptional fusion into the chromosome of strains D33712 and D23580 (Methods; Table S8) to interrogate the expression of the key SPI2 operon with flow cytometry. Fig. 5B shows that in NonSPI2 media, the *ssaG* promoter was expressed at a 62% higher level in D33712 than in D23580 confirming the results of the transcriptomic analysis.

Because only a proportion of *S*. Typhimurium cells express certain pathogenicity island-encoded genes during *in vitro* growth (Hautefort et al. 2003, Ackermann et al. 2008), we determined whether the increased level of expression of SPI2 genes (Fig. 4B) was caused by a higher proportion of D33712 cells expressing SPI2 than D23580 cells. Using derivatives of the two strains that carried the *ssaG*-GFP^+^ construct, we determined the numbers of fluorescent and non-fluorescent cells with flow cytometry (Methods). Under non-inducing conditions, slightly more D37712 cells expressed the *ssaG* SPI2 promoter than D23580 cells (65% vs. 60%, respectively) (Fig. 5C). Although this small difference was statistically significant (*t*-test: *P*<.001, n = 3), it did not account for the 62% increased level of non-induced SPI2 expression seen in Fig. 5B.

SPI2 expression is controlled by a complex regulatory system that operates at both a negative and positive level, involving silencing via H-NS (Lucchini et al. 2006), activation by SlyA and SsrB (Fass and Groisman 2009, Walthers et al. 2011) as well as input from OmpR and Fis under non-inducing conditions (Osborne and Coombes 2011). The mechanistic basis of the aberrant SPI2 expression in strain D37712 is worthy of further study. Possible explanations include the incomplete silencing of SPI2 transcription or the partial activation of the SPI2 virulence genes under non-inducing growth conditions by an unknown regulatory factor.

### Increased fitness of *S*. Typhimurium ST313 sublineage L2.2 compared with L2.0 in minimal media

It has become increasingly clear that distinct *Salmonella* pathovariants have evolved particular phenotypic properties that confer fitness advantages during infection of particular avian or mammalian hosts (Branchu et al. 2018). Because *S*. Typhimurium ST313 L2.2 appeared to have displaced *S*. Typhimurium ST313 L2.0 in Malawi, we speculated that *S*. Typhimurium ST313 L2.2 might have a competitive edge in some situations. Accordingly, we determined bacterial fitness using a mixed-growth competition assay (Wiser and Lenski 2015, Lian et al. 2023). The competitive index was calculated in three different growth media using pair-wise combinations of strains D37712 and D23580. Two independent approaches were used to phenotypically distinguish the two strains, one based on antibiotic resistance (Fig. 5D) and the other based on fluorescent tagging (Fig. S5).

To confirm that strains engineered to be kanamycin-resistant or gentamicin-resistant did not impact on fitness (Methods), we first verified that the tagged variants of D37712 or D23580 did not confer a growth advantage in LB or NonSPI2 media (Fig. S7). Next, we used a mixed-growth assay to investigate fitness of *S*. Typhimurium ST313 L2.0 strain D23580 or *S*. Typhimurium ST313 L2.2 strain D37712 during growth in LB, or InSPI2 or NonSPI2 minimal media. The data show that both strains grew at similar levels following overnight mixed growth in nutrient-rich LB media, but D37712 had a competitive advantage during mixed growth in InSPI2 media (CI = 1.79; *P*<.05) and a greater competitive edge in NonSPI2 media (CI = 2.20; *P*<.0001).

We then used an independent fluorescence-based approach to assess the fitness of strains D23580 and D37712 during mixed growth in NonSPI2 media. This time, the strains were engineered to carry either mScarlet or sGFP2 proteins and the mixed-growth experiments involved pair-wise comparisons of reciprocally tagged strains. The flow cytometric data showed that in both cases D37712 had a significant competitive advantage in NonSPI2 media (Figs. S5 and S6).

This combination of antibiotic resistance-based and fluorescence-based competitive index experiments lead us to conclude that *S*. Typhimurium ST313 L2.2 strain D37712 had a clear fitness advantage over *S*. Typhimurium ST313 L2.0 strain D23580 during mixed growth in two formulations of minimal media. The molecular basis of this fitness advantage remains to be established.

## Discussion

Here, we report that *S*. Typhimurium ST313 L2.0 has been clonally replaced by the ST313 sublineages L2.2 and L2.3 as a cause of bloodstream infection in Blantyre, Malawi. In 2018, L2.2 represented the majority of the ST313 strains isolated from hospitalized patients in Malawi at the QECH. Our comparative genomic analysis of ST313 L2.3 identified 30 chromosomal alterations, one of which generated a deletion of the *sseI* effector gene.

Our RNA-seq-based analysis of ST313 L2.2 involved a detailed comparison versus ST313 L2.0 which revealed a key difference involving SPI2 expression. Following initial observations at the transcriptomic level in the ST313 L2 and L2.2 strains grown in a pH-neutral minimal medium (NonSPI2), the increased expression of SPI2 was confirmed at the single-cell level using an *ssaG* transcriptional fusion.

A series of experiments showed that the ST313 L2.2 strain D37712 had a competitive advantage over L2 strain D23580 during mixed growth in minimal media. We propose that this increased fitness of *S*. Typhimurium ST313 L2.2 has contributed to the replacement of ST313 L2.0 in Malawi in recent years.

Previously, we compared three virulence properties of the *S*. Typhimurium ST313 L2.0 D23580 and ST313 L2.2 D37712 strains. First, experiments involving Mucosal Invariant T (MAIT) cells showed that both D37712 and D23580 fail to elicit the high level of activation of MAIT cells that characterizes infection by *S*. Typhimurium ST19 4/74 (Preciado-Llanes et al. 2020). Second, the D37712 and D23580 strains stimulate similar levels of up-regulation of IL10 gene expression upon infection of human dendritic cells (Aulicino et al. 2022). Third, we showed that both D37712 and D23580 express similarly high levels of the PgtE virulence factor that is responsible for the ability of *S*. Typhimurium ST313 to survive human serum-killing (Hammarlöf et al. 2018). These findings lead us to conclude that the comparative genomic and transcriptomic differences that distinguish *S*. Typhimurium ST313 L2.0 strain D23580 from ST313 L2.2 D37712 (Fig. 4) do not modulate the ability of the pathogens to activate human MAIT cells or dendritic cells or to influence the PgtE-mediated serum survival phenotype of *S*. Typhimurium ST313.

Ideally, the implications of the competitive advantage of ST313 L2.2 would be determined in the context of pathogenesis. However, we lack an informative infection model for *S*. Typhimurium ST313 (Lacharme-Lora et al. 2019), and it is not yet possible to experimentally determine whether the improved fitness of L2.2 significantly enhances the success of ST313 during infection of humans.

We have investigated the intricate interplay of gene function that underpins the success of *S*. Typhimurium ST313 L2.2. It is hoped that our findings will contribute to future therapeutic or prophylactic strategies for combatting iNTS infections in the African setting.

## Supplementary Material

uqae005_Supplemental_File
